# Acquisition of Plasmid with Carbapenem-Resistance Gene *bla*_KPC2_ in Hypervirulent *Klebsiella pneumoniae*, Singapore

**DOI:** 10.3201/eid2603.191230

**Published:** 2020-03

**Authors:** Yahua Chen, Kalisvar Marimuthu, Jeanette Teo, Indumathi Venkatachalam, Benjamin Pei Zhi Cherng, Liang De Wang, Sai Rama Sridatta Prakki, Weizhen Xu, Yi Han Tan, Lan Chi Nguyen, Tse Hsien Koh, Oon Tek Ng, Yunn-Hwen Gan

**Affiliations:** National University of Singapore, Singapore (Y. Chen, Y.H. Tan, L.C. Nguyen, Y.-H. Gan);; National Centre for Infectious Diseases, Singapore (K. Marimuthu, L.D. Wang, S.R.S. Prakki, W. Xu, O.T. Ng);; Tan Tock Seng Hospital, Singapore (K. Marimuthu, O.T. Ng);; National University Hospital, Singapore (J. Teo);; Singapore General Hospital, Singapore (I. Venkatachalam, B.P.Z. Cherng, T.H. Koh);; Nanyang Technological University, Singapore (O.T. Ng)

**Keywords:** *Enterobacteriaceae*, *Klebsiella pneumoniae*, carbapenemase, carbapenem resistance, multidrug resistance, hypervirulent, *bla*_KPC-2_, whole-genome sequencing, genome analysis, plasmid, Singapore, superbug, antimicrobial resistance, bacteria, virulence, hypermucoviscosity, conjugation, K1, K2

## Abstract

The convergence of carbapenem-resistance and hypervirulence genes in *Klebsiella pneumoniae* has led to the emergence of highly drug-resistant superbugs capable of causing invasive disease. We analyzed 556 carbapenem-resistant *K. pneumoniae* isolates from patients in Singapore hospitals during 2010–2015 and discovered 18 isolates from 7 patients also harbored hypervirulence features. All isolates contained a closely related plasmid (pKPC2) harboring *bla*_KPC-2_, a *K. pneumoniae* carbapenemase gene, and had a hypervirulent background of capsular serotypes K1, K2, and K20. In total, 5 of 7 first patient isolates were hypermucoviscous, and 6 were virulent in mice. The pKPC2 was highly transmissible and remarkably stable, maintained in bacteria within a patient with few changes for months in the absence of antimicrobial drug selection pressure. Intrapatient isolates were also able to acquire additional antimicrobial drug resistance genes when inside human bodies. Our results highlight the potential spread of carbapenem-resistant hypervirulent *K. pneumoniae* in Singapore.

The rise of multidrug-resistant (MDR) *Enterobacteriaceae* prompted the World Health Organization to classify carbapenem-resistant *Enterobacteriaceae*, of which *Klebsiella* is the most common genus, on the global priority list of antibiotic-resistant bacteria in 2017 (*1*). Carbapenem-resistant *K. pneumoniae* (CRKP, also including *K. quasipneumoniae*) infections are generally hospital acquired, particularly among elderly and immunocompromised patients (*2*,*3*). The major carbapenemases include *K. pneumoniae* carbapenemase (KPC), New Delhi metallo-β-lactamase, and carbapenem-hydrolyzing class D β-lactamase (OXA), all of which have spread globally (*4*–*7*). 

The Carbapenemase-Producing *Enterobacteriaceae* in Singapore (CaPES) study initiated in 2013 revealed that the rate of incident carbapenem-resistant *Enterobacteriaceae* clinical cultures in government hospitals in Singapore increased during 2011–2013 and plateaued thereafter (*8*). The number of cases of hypervirulent *K. pneumoniae* has increased in the past 3 decades in parts of Asia, and likewise, the number of cases of monomicrobial *Klebsiella*-induced liver abscesses has also increased (*9*,*10*). 

The prevalence of antimicrobial resistance among hypervirulent *K. pneumoniae* isolates is rare compared with that of standard isolates (*11*,*12*); hypervirulent *K. pneumoniae* and CRKP seem to have their own particular reservoirs and remain mostly segregated from each other. However, hypervirulent *K. pneumoniae* and CRKP isolates can converge in the same organism, leading to the emergence of superbugs resistant to antimicrobial drugs of even the last line of treatment that are capable of infecting healthy persons. This emergence has already been reported in China, Brazil, and the United Kingdom (*13*–*15*). The fatal outbreak that occurred in a hospital in China in 2016 was caused by a carbapenem-resistant hypervirulent *K. pneumoniae* strain that had acquired a virulence plasmid by a classic sequence type (ST) 11 strain (*16*). In a study of a collection of >2,200 *K. pneumoniae* genomes, distinct evolutionary patterns of horizontal gene transfer were observed in MDR isolates versus hypervirulent isolates (*17*). The authors of that study postulated that hypervirulent clones might be subject to some sort of constraint against horizontal gene transfer and show more conserved pangenomic diversity than MDR clones. If that hypothesis is correct, MDR clones acquiring virulence genes or *K. pneumoniae* virulence plasmids would be more likely than hypervirulent clones acquiring MDR genes. To investigate this hypothesis, we searched for hypervirulent isolates among 556 CRKP isolates collected at public hospitals of Singapore.

## Materials and Methods

### Bacterial Isolates and Microbiologic Methods

During 2010–2015, all microbiology laboratories in Singapore had been mandated to submit their carbapenem-resistant *Enterobacteriaceae* isolates to the National Public Health Laboratory of Singapore. Using this library, we collected isolates from the CaPES study. We performed species identification, assessed carbapenem resistance, and determined carbapenemase genes as previously described (*8*).

### Whole-Genome Sequencing and Data Analysis

We performed whole-genome sequencing using the MiSeq platform (Illumina, https://www.illumina.com) as previously described (*18*). In addition, we sequenced the complete genomes of 5 isolates from 3 patients, obtaining long reads using the GridION X5 system (Oxford Nanopore Technologies, https://nanoporetech.com) to close the gaps. We de novo assembled the Illumina sequence reads using SPAdes 3.11.1 (*19*) and completed genome assembly using a combination of Illumina and Oxford Nanopore Technologies data with the hybrid assembler Unicycler version 0.4.7 (Appendix Table) (*20*). We deposited whole-genome sequencing data in GenBank (BioProject numbers PRJNA342893, PRJNA557813, and PRJNA591409). We screened genome assemblies for virulence loci and *K. pneumoniae* virulence plasmid–associated loci using kleborate (*21*–*23*). We resolved missing loci and ambiguous alleles by mapping short reads to reference sequences using breseq (*24*) and screened assemblies for antimicrobial resistance genes using ResFinder 3.1 (*25*) and CARD (*26*). We resolved any discrepancies between these 2 gene identifiers by searching blastp (https://blast.ncbi.nlm.nih.gov/Blast.cgi?PAGE=Proteins) using translated gene sequences. We identified plasmid replicons in all completely sequenced genomes using PlasmidFinder with default settings (*27*). For all isolates, we performed core-genome single-nucleotide polymorphism (SNP) analysis against reference genome SGH10 chromosome (GenBank accession no. CP025080) using Parsnp 1.2 (*28*). In plasmid analyses, we generated alignments by mapping assemblies to reference plasmids using bowtie2 (*29*) on the REALPHY server (*30*). We inferred approximate maximum-likelihood phylogenetic trees using FastTree 2 (*31*) and screened completed assemblies for origin of transfer (*oriT*) sites and other transfer-related modules using oriTfinder (*32*).

### Determining Hypermucoviscosity

We assessed hypermucoviscosity of all isolates using the string test (*33*) and a quantitative centrifugation assay (*34*). We used SGH10 as the positive control and SGH10 with *rmpA* deleted as the negative control.

### Mouse Infection

We infected female 7–8-week-old C57BL/6J mice (InVivos, http://www.invivos.com.sg) with 1 × 10^5^ CFU of bacteria diluted in 100 μL phosphate-buffered saline through the intraperitoneal route and assessed for death every 8–16 h. Animal experiments were approved under protocol R18–0252 by the National University of Singapore Institutional Animal Care and Use Committee in accordance with the National Advisory Committee for Laboratory Animal Research guidelines.

### Conjugation Experiments

We measured the transmissibility of the *bla*_KPC-2_–carrying plasmid using a previously described method (*35*). In this experiment, carbapenem-resistant hypervirulent *K. pneumoniae* isolates were the donors and a kanamycin-resistant *Escherichia coli* MG1655 mutant SLC568 strain (*36*) was the recipient. We carried out conjugation on 0.22-µm nitrocellulose filters with donors and recipients incubated at a 1:1 ratio on lysogeny broth (LB) agar plates for 4 h at 37°C. We enumerated transconjugants on LB agar plates containing carbenicillin (100 µg/mL) and kanamycin (50 µg/mL) and recipients on LB agar plates containing kanamycin only. We confirmed transfer of the *bla*_KPC-2_ gene by PCR.

### Antimicrobial Susceptibility Testing

We performed antimicrobial susceptibility testing following the Clinical and Laboratory Standards Institute guidelines. We determined MICs (*37*) and interpreted breakpoints (*38*) of antimicrobial drugs as described.

### Statistical Methods

We performed statistical analyses using GraphPad Prism version 8 (https://www.graphpad.com). We compared samples using the unpaired *t*-test with Welch correction.

## Results

### Discovery of Hypervirulent Features of CRKP

We retrieved 1,312 carbapenem-resistant *Enterobacteriaceae* collected from 6 public hospitals in Singapore during 2010–2015 through the CaPES program and National Public Health Laboratory of Singapore; 1,251 isolates were whole-genome sequenced with Illumina technology, and 556 isolates were *K. pneumoniae*. We searched *K. pneumoniae* isolate genomes for the presence of *K. pneumoniae* virulence plasmid–associated virulence determinants, *rmpA*, *rmpA2*, *iro* (the salmochelin locus), and *iuc* (the aerobactin locus) by using kleborate. We identified 18 isolates (originating from just 7 patients) harboring all of these loci, and 14 of these isolates came from the same 3 patients. We screened the genome assemblies of these 18 isolates for virulence features and compared the characteristics of these isolates with those of 2 known hypervirulent strains, SGH10 (serotype K1 liver abscess–associated isolate from Singapore) (*6*,*39*) and CG43 (serotype K2 clinical isolate from Taiwan) (*40*). We then performed a phylogenetic analysis of the core genomes of all these isolates. 

**Figure 1:**
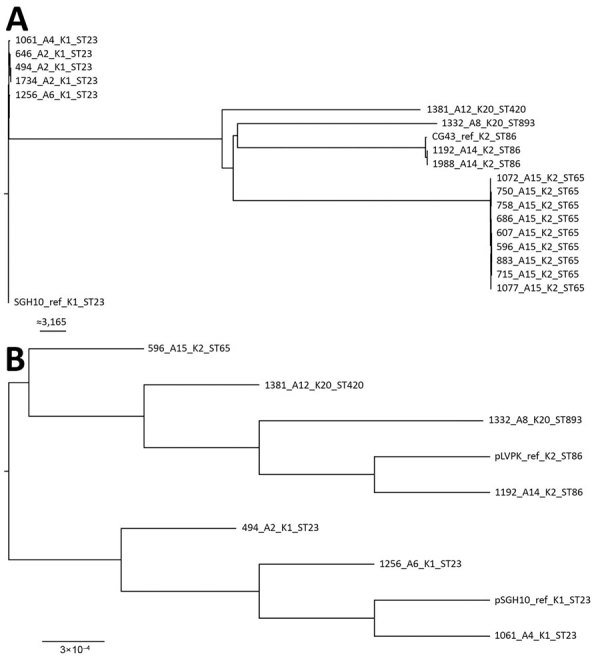
Maximum-likelihood trees of genes from carbapenem-resistant *Klebsiella pneumoniae* isolates, Singapore, 2013–2015. A) Analysis generated using 63,297 single-nucleotide polymorphism sites in the core genome. The chromosomal sequence of SGH10 (GenBank accession no. CP025080) was used as reference. Isolates are closely related to hypervirulent strains SGH10 and CG43. Scale bar indicates number of single-nucleotide polymorphisms. B) Analysis generated from the alignment of *K. pneumoniae* virulence plasmids from the first isolates collected from different patients. The sequences of *K. pneumoniae* virulence plasmid pSGH10 (GenBank accession no. CP025081) was used as reference. Scale bar indicates nucleotide changes per base pair. Trees were drawn using FigTree version 1.4.4 (http://tree.bio.ed.ac.uk/software/figtree) and rooted at the SGH10 branch. Labels indicate isolate no._patient no._K serotype_sequence type. Ref, reference; ST, sequence type.

The differences found among isolates from the same patient were small (0–15 SNPs) (Figure 1, panel A), suggesting that patients with multiple isolates were infected with a single strain. All isolates from patients A2, A4, and A6 were ST23 and serotype K1 (same as SGH10) and, except for ENT1256, carried the same virulence loci as SGH10 (Table 1); ENT1256 had a different allele for *rmpA2*. The core genomes of these isolates were also similar to SGH10, as shown by the phylogenetic analysis (Figure 1, panel A). However, the differences between isolates from patients A2, A4, and A6 were much greater (200–300 SNPs) than the differences between isolates from patient A2 (15 SNPs), indicating that the bacteria from these patients were unlikely to have originated from the same strain. The isolates from patients A14 and A15 were the same serotype (K2) but different sequence types (Table 1), and their core genomes contained many differences (>20,000 SNPs). The isolates from patient A14 were phylogenetically close to the other hypervirulent strain, CG43 (Figure 1, panel A); however, unlike CG43, which carried no yersiniabactin (*ybt*) and colibactin (*clb*) loci, 2 A14 isolates (ENT1192 and ENT1988) had a *ybt* 9 locus on integrative conjugative element *K. pneumoniae* 3 (Table 1). The isolates from the remaining 2 patients (A8 and A12) were the same serotype (K20) but had different sequence types and many core genome differences (>20,000 SNPs). ENT1332 had an unknown *ybt* locus, and ENT1381 had a *ybt* 9 locus on integrative conjugative element *K. pneumoniae* 3. Both of these isolates did not have the *clb* locus. All isolates had hypervirulence backgrounds.

**Table 1:** Classifications and virulence characteristics of 18 carbapenem-resistant *Klebsiella pneumoniae* isolates harboring KPVP-associated genes, Singapore, 2013–2015*

Isolate	Patient no.	K locus	ST	Virulence score	Locus					
					Yersiniabactin	Colibactin	Aerobactin	Salmochelin	*rmpA*	*rmpA2*
ENT494	A2	1	23	5	*ybt* 1, ICEKP10	*clb* 2	*iuc* 1	*iro* 1	2(KPVP-1)	6
ENT646	A2	1	23	5	*ybt* 1, ICEKP10	*clb* 2	*iuc* 1	*iro* 1	2(KPVP-1)	6
ENT1734	A2	1	23	5	*ybt* 1, ICEKP10	*clb* 2	*iuc* 1	*iro* 1	2(KPVP-1)	6
ENT1061	A4	1	23	5	*ybt* 1, ICEKP10	*clb* 2	*iuc* 1	*iro* 1	2(KPVP-1)	6
ENT1256	A6	1	23	5	*ybt* 1, ICEKP10	*clb* 2	*iuc* 1	*iro* 1	2(KPVP-1)	5
ENT1332	A8	20	893	4	*ybt* unknown	–	*iuc* 1	*iro* 1	1(KPVP-1)	2
ENT1381	A12	20	420	4	*ybt* 9, ICEKP3	–	*iuc* 1	*iro* 1	1(KPVP-1)	5
ENT1192	A14	2	86	4	*ybt* 9, ICEKP3	–	*iuc* 1	*iro* 1	2(KPVP-1)	6
ENT1988	A14	2	86	4	*ybt* 9, ICEKP3	–	*iuc* 1	*iro* 1	2(KPVP-1)	6
ENT596	A15	2	65	5	*ybt* 17, ICEKP10	*clb* 3	*iuc* 1	*iro* 1	2(KPVP-1)	6
ENT607	A15	2	65	5	*ybt* 17, ICEKP10	*clb* 3	*iuc* 1	*iro* 1	2(KPVP-1)	6
ENT686	A15	2	65	5	*ybt* 17, ICEKP10	*clb* 3	*iuc* 1	*iro* 1	2(KPVP-1)	6
ENT715	A15	2	65	5	*ybt* 17, ICEKP10	*clb* 3	*iuc* 1	*iro* 1	2(KPVP-1)	6
ENT750	A15	2	65	5	*ybt* 17, ICEKP10	*clb* 3	*iuc* 1	*iro* 1	2(KPVP-1)	6
ENT758	A15	2	65	5	*ybt* 17, ICEKP10	*clb* 3	*iuc* 1	*iro* 1	2(KPVP-1)	6
ENT883	A15	2	65	5	*ybt* 17, ICEKP10	*clb* 3	*iuc* 1	*iro* 1	2(KPVP-1)	6
ENT1072	A15	2	65	5	*ybt* 17, ICEKP10	*clb* 3	*iuc* 1	*iro* 1	2(KPVP-1)	6
ENT1077	A15	2	65	5	*ybt* 17, ICEKP10	*clb* 3	*iuc* 1	*iro* 1	2(KPVP-1)	6
ENT495†		66	841	0	–	–	–	–	–	–
SGH10‡		1	23	5	*ybt* 1, ICEKP10	*clb* 2	*iuc* 1	*iro* 1	2(KPVP-1)	6
CG43§		2	86	3	–	–	*iuc* 1	*iro* 1	1(KPVP-1)	1

*ICEKP, integrative conjugative element *K. pneumoniae*; KPVP, *K. pneumoniae* virulence plasmid; ST, sequence type. †Control carbapenem-resistant *K. pneumoniae* isolate (classified as *K. quasipneumoniae* by GenBank). ‡GenBank accession nos. CP025080 and CP025081.  §GenBank accession nos. CP006648 and AY378100.

We also performed a whole-genome phylogenetic analysis on the *K. pneumoniae* virulence plasmid carried in the first isolates obtained from all patients, using the *K. pneumoniae* virulence plasmid sequence from SGH10 (pSGH10) as reference. The *K. pneumoniae* virulence plasmids appeared to form 2 separate clades, 1 for all K1 isolates and the other for K2 and K20 isolates (Figure 1, panel B).

**Figure 2:**
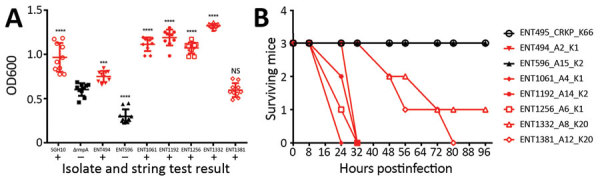
Hypervirulence assessment of first isolates from 7 patients with CRKP infections, Singapore, 2013–2015. A) Hypermucoviscosity of isolates as indicated by a low-speed centrifugation assay and the string test. For the centrifugation assay, *Klebsiella pneumoniae* isolates were grown in Luria broth overnight at 37°C and centrifuged (10 minutes at 2,000 × *g*), and OD600s of supernatants were measured. Each symbol represents the value for an individual clone (n = 10) from 3 independent experiments. Horizontal bars indicate means and error bars SDs. For the string test, *K. pneumoniae* were grown on sheep blood agar (2 days at 37°C). Red indicates a positive string test result. B) In vivo virulence in mice. Female C57BL/6J mice (7–8 weeks old, 3 mice/isolate) were injected with 1 × 10^5^ CFU of bacteria by the intraperitoneal route. Every 8 or 16 hours, mice were checked and scored for death. If necessary, they were euthanized and counted as dead. The experiment was stopped at 96 hours postinfection. For each isolate, patient number and K serotype is indicated. CRKP, carbapenem-resistant *Klebsiella pneumoniae*; OD, optical density; NS, not significant. ***p = 0.0001; ****p<0.0001.

The virulence potentials of all isolates were high (Table 1); the virulence scores predicted by kleborate for all isolates (>4) were higher than the score (3) predicted for the hypervirulent K2 reference strain CG43. Hypervirulent *K. pneumoniae* isolates are generally defined as carrying *K. pneumoniae* virulence plasmid–associated loci and having a hypermucoviscous phenotype (including a positive string test result), which is dependent on regulator RmpA (*40*,*41*). We measured the hypermucoviscosity of the first isolates from all patients using both the string test and a centrifugation assay; 6 of 7 isolates formed strings, and 5 of 7 isolates were hypermucoviscous according to the centrifugation assay (Figure 2, panel A). Only ENT596 was negative by both tests. 

The virulence potential of the carbapenem-resistant hypervirulent *K. pneumoniae* isolates was further determined in an intraperitoneal mouse infection model. The first isolates from 6 of 7 patients killed >50% of the infected mice within 96 hours; only ENT596 (like control isolate ENT495, a CRKP strain carrying pKPC2 but not hypervirulent) did not kill any mice (Figure 2, panel B). Serotype K1 isolates were the most virulent, and serotype K20 isolates took longer to kill. The virulence of ENT596 did not correlate with its predicted score; this isolate demonstrated concurrent loss of hypermucoviscosity and virulence in mice (Table 1; Figure 2), which might have been caused by the loss of expression of the virulence genes. The average length of hospitalization of the 7 patients harboring these isolates was 97 days, which was much longer than the average length of stay for 249 patients with carbapenem-resistant *Enterobacteriaceae* infection (38 days) (*8*). Taking into account all our evidence, we conclude that the isolates from 6 of 7 patients (A2, A4, A6, A8, A12, and A14) are phenotypically hypervirulent, and the isolates from patient A15 are phenotypically nonhypervirulent (although these isolates have a hypervirulent genetic background).

### Highly Conserved Plasmid Harboring *bla*_KPC-2_ on All Carbapenem-Resistant Hypervirulent *K. pneumoniae* Isolates 

We screened the assemblies of all 18 carbapenem-resistant hypervirulent *K. pneumoniae* isolates for acquired antimicrobial resistance genes using ResFinder (*25*) and CARD (*26*). Except for endogenous penicillin resistance, hypervirulent *K. pneumoniae* isolates are generally considered to be susceptible to antimicrobial drugs. Our search results showed that SGH10 harbored the β-lactam resistance gene *bla*_SHV-11_ and resistance genes against fluoroquinolones (*oqxA* and *oqxB*) and fosfomycins (*fosA6*) (Table 2). In total, 17 of 18 isolates carried these 3 resistance genes; 1 isolate from patient A15 (ENT686) did not have the *oqxA* and *oqxB* genes, and these genes were also not detectable by PCR. All isolates carried an identical set of the following 4 genes: *bla*_KPC-2_, *bla*_TEM-1A_, *bla*_TEM-1B_, and *mph*(A). In the 5 completely sequenced genomes (ENT494, ENT646, and ENT1734 [patient A2]; ENT1192 [patient A14]; and ENT607 [patient A15]; Appendix Table), we located these 4 genes on a 71,861-bp plasmid, which we named pKPC2. The sequences of the pKPC2s in the 4 isolates from patients A2 and A14 were identical. The pKPC2 in ENT607 from patient A15 had only 1-bp difference. 

**Table 2:** Isolation date, sampling site, and resistance genes of carbapenem-resistant hypervirulent *Klebsiella pneumoniae* isolates, Singapore, 2013–2015

Isolate	Patient no.	Date of isolation	Sampling site	β-lactam resistance genes	Other resistance genes
ENT494	A2	2013 Jun 7	Sputum	*bla*_SHV-11_, *bla*_KPC-2_, *bla*_TEM-1A_, *bla*_TEM-1B_	*oqxA*, *oqxB*, *fosA6*, *mph*(A)
ENT646	A2	2013 Sep 18	Blood	*bla*_SHV-11_, *bla*_KPC-2_, *bla*_TEM-1A_, *bla*_TEM-1B_, *bla*_OXA-1_	*oqxA*, *oqxB*, *fosA6, mph*(A), *qnrB1*, *aac(6')-lb-cr, catB3*, *dfrA14*
ENT1734	A2	2014 Dec 19	Rectum	*bla*_SHV-11_, *bla*_KPC-2_, *bla*_TEM-1A_, *bla*_TEM-1B_, *bla*_OXA-1_	*oqxA*, *oqxB*, *fosA6*, *mph*(A), *catB3*, *aac(6')-lb-cr*
ENT1061	A4	2014 Mar 13	Blood	*bla*_SHV-11_, *bla*_KPC-2_, *bla*_TEM-1A_, *bla*_TEM-1B_	*oqxA*, *oqxB*, *fosA6*, *mph*(A)
ENT1256	A6	2014 Jun 20	Rectum	*bla*_SHV-11_, *bla*_KPC-2_, *bla*_TEM-1A_, *bla*_TEM-1B_	*oqxA*, *oqxB*, *fosA6*, *mph*(A)
ENT1332	A8	2014 Jul 13	Rectum	*bla*_SHV-1_, *bla*_KPC-2_, *bla*_TEM-1A_, *bla*_TEM-1B_, *bla*_CTX-M-15_	*oqxA*, *oqxB*, *fosA6*, *mph*(A), *aac(6')-lb-cr*, *aadA16*, *qnrB6*, *arr-3*, *sul1*, *tet*(A), *dfrA27*
ENT1381	A12	2014 Aug 10	Midstream urine	*bla*_SHV-75_, *bla*_KPC-2_, *bla*_TEM-1A_, *bla*_TEM-1B_, *bla*_OXA-1_	*oqxA*, *oqxB*, *fosA6*, *mph*(A)
ENT1192	A14	2014 May 24	Rectum	*bla*_SHV-1_, *bla*_KPC-2_, *bla*_TEM-1A_, *bla*_TEM-1B_, *bla*_OXA-1_	*oqxA*, *oqxB*, *fosA5*, *mph*(A), *aac(6')-lb-cr*, *qnrB1*, *catB3*, *tet*(A), *dfrA14*
ENT1988	A14	2015 Apr 16	Feces, rectum	*bla*_SHV-1_, *bla*_KPC-2_, *bla*_TEM-1A_, *bla*_TEM-1B_, *bla*_OXA-1_	*oqxA*, *oqxB*, *fosA5*, *mph*(A), *aac(6')-lb-cr*, *qnrB1*, *catB3*, *tet*(A), *dfrA14*
ENT596	A15	2013 Aug 22	Urine	*bla*_SHV-11_, *bla*_KPC-2_, *bla*_TEM-1A_, *bla*_TEM-1B_	*oqxA*, *oqxB*, *fosA6*, *mph*(A)
ENT607	A15	2013 Aug 22	Sputum	*bla*_SHV-11_, *bla*_KPC-2_, *bla*_TEM-1A_, *bla*_TEM-1B_	*oqxA*, *oqxB*, *fosA6*, *mph*(A), *aadA1*, *cmlA1*, *arr-2*, *sul1*
ENT686	A15	2013 Oct 4	Tracheostomy aspirate	*bla*_SHV-11_, *bla*_KPC-2_, *bla*_TEM-1A_, *bla*_TEM-1B_	*fosA6, mph*(A)
ENT715	A15	2013 Oct 17	Trachea aspirate	*bla*_SHV-11_, *bla*_KPC-2_, *bla*_TEM-1A_, *bla*_TEM-1B_	*oqxA*, *oqxB*, *fosA6*, *mph*(A)
ENT750	A15	2013 Oct 31	Blood	*bla*_SHV-11_, *bla*_KPC-2_, *bla*_TEM-1A_, *bla*_TEM-1B_	*oqxA*, *oqxB*, *fosA6*, *mph*(A)
ENT758	A15	2013 Oct 31	Tracheal aspirate	*bla*_SHV-11_, *bla*_KPC-2_, *bla*_TEM-1A_, *bla*_TEM-1B_	*oqxA*, *oqxB*, *fosA6*, *mph*(A)
ENT883	A15	2013 Dec 14	Sputum	*bla*_SHV-11_, *bla*_KPC-2_, *bla*_TEM-1A_, *bla*_TEM-1B_	*oqxA*, *oqxB*, *fosA6*, *mph*(A)
ENT1072	A15	2014 Mar 26	Sputum	*bla*_SHV-11_, *bla*_KPC-2_, *bla*_TEM-1A_, *bla*_TEM-1B_	*oqxA*, *oqxB*, *fosA6*, *mph*(A)
ENT1077	A15	2014 Mar 28	Rectum	*bla*_SHV-11_, *bla*_KPC-2_, *bla*_TEM-1A_, *bla*_TEM-1B_	*oqxA, oqxB*, *fosA6*, *mph*(A), *tet*(A), *dfrA14*
ENT495*		2013 Jun 8	Not known	*bla*_OKP-B-6_, *bla*_KPC-2_, *bla*_TEM-1A_, *bla*_TEM-1B_, *bla*_OXA-1_	*oqxA*, *oqxB*, *fosA6*, *mph*(A), *tet*(A), *dfrA14*, *aac(6')-Ib-cr*, *aadA1*, *arr-2*, *cmlA5*, *ereA*, *qnrB1*, *sul1*
SGH10		Not applicable	Blood	*bla*_SHV-11_	*oqxA*, *oqxB*, *fosA6*

*Control carbapenem-resistant *K. pneumoniae* isolate (classified as *K. quasipneumoniae* by GenBank).

**Figure 3:**
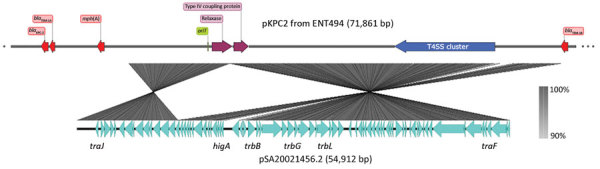
Main features of pKPC2 from *Klebsiella pneumoniae* isolate ENT494, Singapore, 2013, and comparison with pSA20021456.2. Image was generated by using SnapGene Viewer (https://www.snapgene.com) and Easyfig (https://github.com/mjsull/Easyfig). KPC, *Klebsiella pneumoniae* carbapenemase; *oriT*, origin of transfer; T4SS, type IV secretion system.

A blastn search for pKPC2 revealed that the highest hit was a plasmid from *Salmonella enterica* (pSA20021456.2, GenBank accession no. CP030221), which covered 74% of pKPC2 with >99% identity (Figure 3). No attribute information was available for the *Salmonella* strain, and pSA20021456.2 carries no antimicrobial drug resistance genes. No incompatibility groups were detected on either plasmid by PlasmidFinder. 

**Figure 4:**
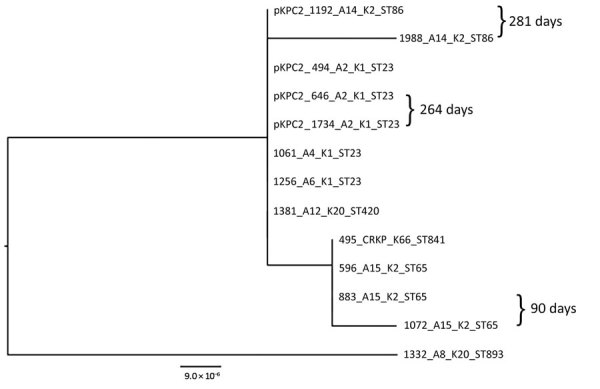
Maximum-likelihood analysis of pKPC2 plasmids from carbapenem-resistant hypervirulent *Klebsiella pneumoniae* isolates, Singapore, 2013–2015. pKPC2_494 was used as reference. Labels indicate isolate no._patient no._K serotype_sequence type. Days between isolate collection are indicated. Scale bar indicates nucleotide changes per base pair. KPC, *Klebsiella pneumoniae* carbapenemase; ST, sequence type.

We performed a phylogenetic analysis of the pKPC2 sequences carried in the first isolates and other select isolates from all 7 patients, including the pKPC2 from ENT494 (i.e., pKPC2_494) as reference (Figure 4). This analysis revealed that all isolates carried sequences almost identical to pKPC2_494 (coverage and identity >99%). Because all isolates carried the 4-gene set, they probably had a plasmid closely related to pKPC2_494. Three patients had multiple isolates with pKPC2. The clinical records show long time-interval gaps of antimicrobial drug nonexposure between some isolates. The comparison also shows that the pKPC2-related plasmids were remarkably stable; they were maintained in bacteria with few changes for 90–281 days in the patients not undergoing antimicrobial drug treatment (Figure 4).

**Figure 5:**
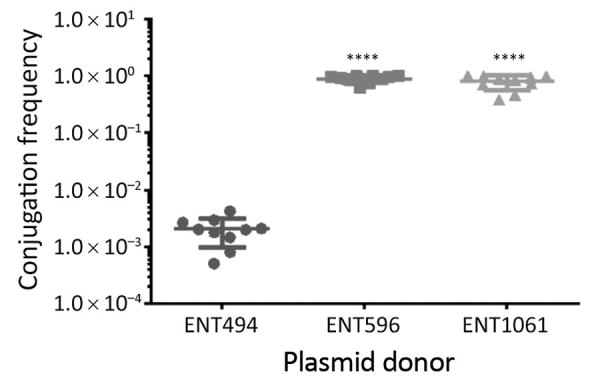
Conjugation ability of pKPC2 from carbapenem-resistant hypervirulent *Klebsiella pneumoniae* isolates, Singapore, 2013–2014, to *Escherichia coli* SLC568. Filter matings were performed for 4 h. The conjugation frequency is the number of CFUs of transconjugants divided by the number of CFUs of recipients. Each symbol represents the value for an individual clone (n = 10) from 3 independent experiments. Horizontal bars indicate means and error bars SD. KPC, *Klebsiella pneumoniae* carbapenemase. ****p<0.0001.

Besides resistance genes, pKPC2 had a complete set of conjugative machinery with all 4 essential modules (*oriT*, relaxase, type IV coupling protein, and a type IV secretion system cluster), suggesting the plasmid is self-transmissible. The *Salmonella* plasmid pSA20021456.2 has a relaxase, type IV coupling protein, and type IV secretion system cluster similar to pKPC2 but no *oriT* site. We selected 3 isolates that had only 1 antimicrobial drug resistance plasmid to assess the transmissibility of pKPC2. We performed filter mating on LB agar using the kanamycin-resistant *E. coli* strain SLC568 as the recipient. After 4 hours of incubation, ≈80% of recipients (≈0.8 × 10^0^ transconjugate/recipient) had received pKPC2 from ENT596 and ENT1061 (Figure 5). The conjugation frequency was ≈0.2% (≈2.0 × 10^–4^ transconjugate/recipient) when ENT494 was the donor. We confirmed the transconjugants acquired the *bla*_KPC-2_ gene by PCR (using 10 colonies for each donor). K serotype, sequence type, and hypermucoviscosity of donors could not explain the observed differences in conjugation efficiency.

To assess the role of pKPC2 in conferring antimicrobial drug resistance to host strains, we measured the MICs of antimicrobial drugs against the 3 isolates from patient A2 and compared with SGH10 (Table 3). SGH10 was resistant to penicillins and fosfomycin but susceptible to the 2 fluroquinolones tested, even though the strain carried the fluroquinolone efflux pump genes *oqxA* and *oqxB* (Table 2). This finding is consistent with the low *oqxB* expression seen for most *K. pneumoniae* strains (*42*). SGH10 was susceptible to cephems and carbapenems. In contrast, all 3 isolates from patient A2 were resistant to ceftriaxone, imipenem, and meropenem (Table 3), most likely because of the presence of pKPC2. These data show that pKPC2 is a highly transmissible plasmid that confers resistance to all 3 types of β-lactams.

**Table 3:** MICs of antimicrobial drugs against 3 carbapenem-resistant hypervirulent *Klebsiella pneumoniae* isolates from patient A2, Singapore, 2013–2014, compared with reference strain SGH10*

Antimicrobial drug group and drug	SGH10	ENT494	ENT646	ENT1734
Penicillins				
Ampicillin	**>64**	**>64**	**>64**	**>64**
Piperacillin	**32**	**>64**	**>64**	**>64**
Cephems				
Ceftriaxone	<1	**>64**	**>64**	**>64**
Carbapenems				
Imipenem	<1	**>64**	**>64**	**>64**
Meropenem	<1	**8**	**16**	**8–16**
Aminoglycosides				
Amikacin	<1	<1	2–4	4–8
Gentamicin	<1	<1	<1	<1
Kanamycin	2	4	**16**	**32**
Tetracyclines				
Doxycycline	<1	2	2	2
Fluoroquinolones				
Ciprofloxacin	<1	<1	**8**	<1
Levofloxacin	<1	<1	<1	<1
Folate pathway antagonists				
Sulfamethoxazole	128	**512**	**512**	**>512**
Trimethoprim	<1	<1	**>64**	<1
Phenicols				
Chloramphenicol	4	4–8	4–8	4
Fosfomycins				
Fosfomycin	**>64**	**>64**	**>64**	**>64**
Lipopeptides				
Colistin	4	2	2–4	2–4
Polymyxin B	4	4	4	4

*Bold numbers indicate resistance as interpreted by Clinical and Laboratory Standards Institute interpretative criteria for MICs (*38*).

### Within-Patient Microevolution of Carbapenem-Resistant Hypervirulent *K. pneumoniae* Isolates

**Figure 6:**
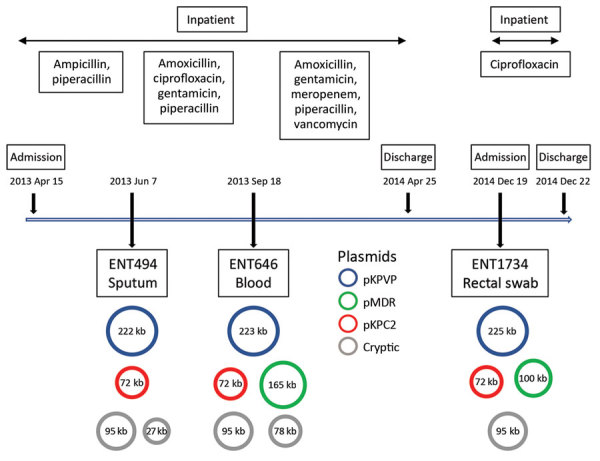
Timeline showing antimicrobial drug exposure and plasmid changes in 3 carbapenem-resistant hypervirulent *Klebsiella pneumoniae* isolates from patient A2, Singapore, 2013–2014. pMDR646 contains genes *aac(6’)-lb-cr*,*bla*_OXA-1_, *qnrB1*, *catB3*, and *dfrA14*. pMDR1734 contains genes *aac(6’)-lb-cr*, *bla*_OXA-1_, and *catB3*. pKPC2 contains genes *bla*_KPC-2_, *bla*_TEM-1A_, *bla*_TEM-1B_, and *mph*(A). KPC, *Klebsiella pneumoniae* carbapenemase; KPVP, *Klebsiella pneumoniae* virulence plasmid; MDR, multidrug resistance.

Using multiple isolates from the same patient, we set out to determine the changes that occurred in the genome of 1 carbapenem-resistant hypervirulent *K. pneumoniae* population over the course of an infection. This analysis enabled us to track the carriage of genes conferring carbapenem resistance and MDR in the bacteria versus antimicrobial drug exposure over time. With the available clinical data from patient A2, we reconstructed a timeline of the evolution of the 3 isolates from this patient, showing their plasmid content and antimicrobial drug exposure (Figure 6). 

In addition to the chromosome, *K. pneumoniae* virulence plasmid, and pKPC2, all 3 isolates from patient A2 carried 2 or 3 additional plasmids (Appendix Table). Isolates were of the same infecting strain but lost or gained MDR plasmids over time. The *K. pneumoniae* virulence plasmid and pKPC2 were stable, showing few changes over a year. Treatment with gentamicin and ciprofloxacin correlated with the appearance of an MDR plasmid in ENT646 that encoded resistance genes to both classes of antimicrobial drugs. This 165-kb MDR plasmid (named pMDR646) carried *aac*(6')*-lb-cr*, *bla*_OXA-1_, *qnrB1*, *catB3*, and *dfrA14*. In the third isolate from patient A2 (ENT1734), the MDR plasmid downsized to 100 kb (pMDR1734) and carried only *aac*(6')*-lb-cr*, *bla*_OXA-1_, and *catB3*. The downsizing appeared to be the result of extensive deletion and reorganization (Appendix Figure). Antimicrobial susceptibility testing data also showed that this bacterium was resistant to fewer antimicrobial drugs; isolate ENT646 was resistant to 7 classes of antimicrobial drugs, and ENT1734 was resistant to 6 classes of drugs (Table 3). Both ENT646 and ENT1734 carried the broad-range resistance gene *aac(6')-lb-cr*, which confers resistance against aminoglycoside and fluoroquinolone antimicrobial drugs. This gene was likely responsible for the observed resistance to kanamycin, and *qnrB1* (in ENT646) was likely responsible for resistance to ciprofloxacin (Table 3). Both isolates were also resistant to sulfamethoxazole, which we could not attribute to any resistance gene or mutation. All 3 isolates carried a 95-kb plasmid with unknown virulence or resistance attributes (Figure 6). The sizes of the chromosome and *K. pneumoniae* virulence plasmid gradually increased over time, mainly through the acquisition of mobile genetic elements.

Nine isolates were collected from patient A15 at various time points and from different anatomic sites (Table 2); all were identical in their core genomes (0 SNP differences). Two A15 isolates acquired antimicrobial drug resistance genes in addition to those on pKPC2; 1 of the first isolates, ENT607, had *aadA1*, *mph*(A), *cmlA1*, *arr-2*, and *sul1*, which were all on a 105-kb plasmid (named pMDR607). This plasmid appeared unstable and was absent in later isolates. The last isolate, ENT1077, had acquired tetracycline and trimethoprim resistance genes not seen in the other isolates.

## Discussion

We report the coexistence of hypervirulence and carbapenem resistance within the same *K. pneumoniae* isolates in Singapore. These isolates dated back to 2013; however, their existence could have occurred even earlier because collection started in 2010. All carbapenem-resistant hypervirulent *K. pneumoniae* isolates in this study harbored a *bla*_KPC-2_ gene. In studies conducted in China, most patients infected with carbapenem-resistant hypervirulent *K. pneumoniae* also carried the *bla*_KPC-2_ gene (*16*,*43*–*45*). We and others have described hypervirulent *K. pneumoniae* in community-acquired liver abscesses in Singapore, where most isolates are capsular serotypes K1 and K2 (*6*,*46*). Most (>80%) liver abscess isolates are estimated to belong to sublineage CG23-I (includes reference strain SGH10) (*39*). The carbapenem-resistant hypervirulent *K. pneumoniae* isolates from 5 of 7 patients in this study were K1 or K2 serotype and 6 of 7 isolates were highly virulent, as predicted. Our results suggest that pKPC2 can be stably maintained in a hypervirulent *K. pneumoniae* bacterial host. Thus, the possible dissemination of the *bla*_KPC-2_ gene to hypervirulent strains present in a carriage state within communities is a concern.

In a study in China, 1,838 isolates were analyzed, and 21 carbapenem-resistant hypervirulent *K. pneumoniae* isolates were found and classified as ST11, ST65, ST268, ST595, and ST692 (*44*). In another report, 5 ST11 CRKP isolates were documented as having acquired *K. pneumoniae* virulence plasmids (*16*). In these reports, a *K. pneumoniae* virulence plasmid or parts of one was probably co-opted into the prevalent CRKP strain in that region, which was an ST11 strain carrying the *bla*_KPC-2_ gene. According to our previous study, only 5% of CRKP isolates in Singapore were ST11 (*8*). However, in another study, 7 carbapenem-resistant hypervirulent *K. pneumoniae* isolates were identified, 4 of which were ST23 and serotype K1 (*47*). In 2 case studies, ST23 (hypervirulent) isolates were reported to have acquired carbapenem-resistance genes (*14*,*48*). All our isolates in this investigation had the hypervirulence background and acquired carbapenem-resistance and MDR genes; the MDR genes appeared to move in and out of the parental strain over time within the patient. Our observations are consistent with earlier reports in China involving ST65 isolates, which were likely hypervirulent, indicating that hypervirulent isolates can acquire antimicrobial drug resistance. Therefore, whether a superbug is more likely to arise from a carbapenem-resistant isolate acquiring a *K. pneumoniae* virulence plasmid or some of its genes versus a hypervirulent isolate acquiring MDR genes is unclear. Both ways of exchange appear possible, perhaps depending on the prevalence of the circulating sequence type in a particular setting and in vivo selection pressures.

We also show that, although isolates from different patients were similar in terms of the virulence and carbapenem-resistance plasmids, these isolates do not arise from transmission events. For patients with multiple isolates, the core genomes were highly conserved, suggesting a single infecting isolate gained or lost various antimicrobial drug resistance genes over time. For patient A2, our longitudinal data suggest that the use of distinct antimicrobial drugs drove the acquisition of resistance, though we have no direct proof of cause and effect. Of note, in patient A15, the rectal isolate had additional antimicrobial drug resistance, perhaps reflecting that the colon might serve as a reservoir where genetic exchange can take place. Six of the 18 isolates, including the last acquired isolates from the 3 patients with multiple isolates, were collected from feces or rectal swab specimens, suggesting that the intestines are a likely reservoir for persistent carriage. Last, we demonstrated as a proof-of-principle that pKPC2 can be transferred from the *K. pneumoniae* isolates to *E. coli* in vitro with high efficiency. Determining the particular characteristics of pKPC2 that make it so transmissible and stable, particularly during interactions with hypervirulent isolates, and how pKPC2 is acquired by the recipient is essential.

Therefore, this report serves to alert infectious disease clinicians to the possible presence of hypervirulence in MDR or carbapenem-resistant colonizing isolates; patients harboring these isolates are at risk of developing invasive infections. With timely (rather than retrospective) whole-genome sequencing of bacteria, identifying patients harboring hypervirulent and multidrug or carbapenem-resistant isolates at high risk for death should be possible. These patients can be selected for appropriate infection control measures, treatment, and close monitoring. Developing strategies to decolonize the gastrointestinal tracts of patients with such isolates would help minimize the release of these potential superbugs into the community.

Appendix More information about acquisition of plasmid with carbapenem-resistance gene *bla*_KPC-2_ in hypervirulent *Klebsiella pneumoniae*, Singapore.
