# Supplementary material for: Acquisition of Plasmid with Carbapenem-Resistance Gene blaKPC2 in Hypervirulent Klebsiella pneumoniae, Singapore
Source: Emerg Infect Dis. 2020 Mar;26(3):549–59. doi: 10.3201/eid2603.191230 (PMC7045839; doi:10.3201/eid2603.191230)
Supplement: Appendix — More information about acquisition of plasmid with carbapenem-resistance gene blaKPC-2 in hypervirulent Klebsiella pneumoniae, Singapore. [file 19-1230-Techapp-s1.pdf]

Article DOI: https://doi.org/10.3201/eid2603.191230

Acquisition of Carbapenem-Resistance Gene *bla*KPC-2 in Hypervirulent, *Klebsiella pneumoniae*, Singapore

Appendix

Appendix Table. Features of complete genomes*

Isolate/ subject, Replicon, Accession number	Size (bp)	Incompatibility group	oriT	Self-transmissible modules	Features
ENT494 / A2					
Chromosome_494, VONF01000001.1	5443212	-	N	3/4	*bla*$_{SHV-11}$, *oqxA, oqxB, fosA6*
pKpVp_494, VONF01000002.1	221812	IncHI1B	N	1/4	aerobactin and salmochelin loci, *rmpA* genes
pENT494_95kb, VONF01000003.1	95210	IncFIA	Y	4/4	cryptic
pKPC2_494, VONF01000004.1	71861	Not identified	Y	4/4	*bla*$_{KPC-2}$, *bla*$_{TEM-1A}$, *bla*$_{TEM-1B}$, *mph(A)*
pENT494_27kb, VONF01000005.1	27285	Not identified	Y	2/4	cryptic
ENT646 / A2					
Chromosome_646, VONE01000001.1	5444482	-	N	3/4	*bla*$_{SHV-11}$, *oqxA, oqxB, fosA6*
pKpVP_646, VONE01000002.1	223140	IncHI1B	N	1/4	aerobactin and salmochelin loci, *rmpA* genes; pKpVP_494 +1 *IS110*
pMDR_646, VONE01000003.1	165414	IncFIB, IncFII	Y	4/4	*aac(6')-Ib-cr, bla*$_{OXA-1}$, *qnrB1, catB3, dfrA14*
pENT646_95kb, VONE01000004.1	95210	IncFIA	Y	4/4	cryptic; identical to pENT494_95kb
pENT646_78kb, VONE01000005.1	78288	Not identified	N	1/4	cryptic
pKPC2_646, VONE01000006.1	71861	Not identified	Y	4/4	*bla*$_{KPC-2}$, *bla*$_{TEM-1A}$, *bla*$_{TEM-1B}$, *mph(A);* identical to pKPC2_494
ENT1734 / A2					
Chromosome_1734, VOND01000001.1	5448145	-	N	3/4	*bla*$_{SHV-11}$, *oqxA, oqxB, fosA6*
pKpVP_1734, VOND01000002.1	224835	IncHI1B	N	1/4	aerobactin and salmochelin loci, rmpA genes; pKpVp + two *IS110*
pMDR_1734, VOND01000003.1	99794	IncFIB	Y	3/4	*aac(6')-Ib-cr, bla*$_{OXA-1}$, *catB3*; multiple deletions of pMDR_646
pENT1734_95kb, VOND01000004.1	95210	IncFIA	Y	4/4	cryptic; identical to pENT494_95kb
pKPC2_1734, VOND01000005.1	71861	Not identified	Y	4/4	*bla*$_{KPC-2}$, *bla*$_{TEM-1A}$, *bla*$_{TEM-1B}$, *mph(A);* identical to pKPC2_494
ENT1192 / A14					
Chromosome_1192, VOMY01000001.1	5246080	-	Y	2/4	*bla*$_{SHV-1}$, *oqxA, oqxB, fosA5*
pKpVP_1192, VOMY01000002.1	215341	IncHI1B	N	1/4	aerobactin and salmochelin loci, *rmpA* genes
pMDR_1192, VOMY01000003.1	109672	IncFII(K)	Y	4/4	*aac(6')-Ib-cr, qnrB1, catB3, tet(A), dfrA14*
pENT1192_81kb, VOMY01000004.1	81371	IncFIA	N	3/4	cryptic
pKPC2_1192, VOMY01000005.1	71861	Not identified	Y	4/4	*bla*$_{KPC-2}$, *bla*$_{TEM-1A}$, *bla*$_{TEM-1B}$, *mph(A);* identical to pKPC2_494
ENT607 / A15					
Chromosome_607, VOMV01000001.1	5416548	-	N	3/4	*bla*$_{SHV-11}$, *oqxA, oqxB, fosA6*
pKpVP_607, VOMV01000002.1	235891	IncHI1B	N	1/4	aerobactin and salmochelin loci, *rmpA* genes
pMDR_607, VOMV01000003.1	104712	IncFIB, IncFII(K)	Y	4/4	*aadA1, cmlA1, arr-2, sul1*
pKPC2_607, VOMV01000004.1	71861	Not identified	Y	4/4	*bla*$_{KPC-2}$, *bla*$_{TEM-1A}$, *bla*$_{TEM-1B}$, *mph(A);*1 bp difference from pKPC2_494

*KPC, *Klebsiella pneumoniae* carbapenemase; KpVP, *K. pneumoniae* virulence plasmid; MDR, multidrug resistance; N, no; Y, yes.

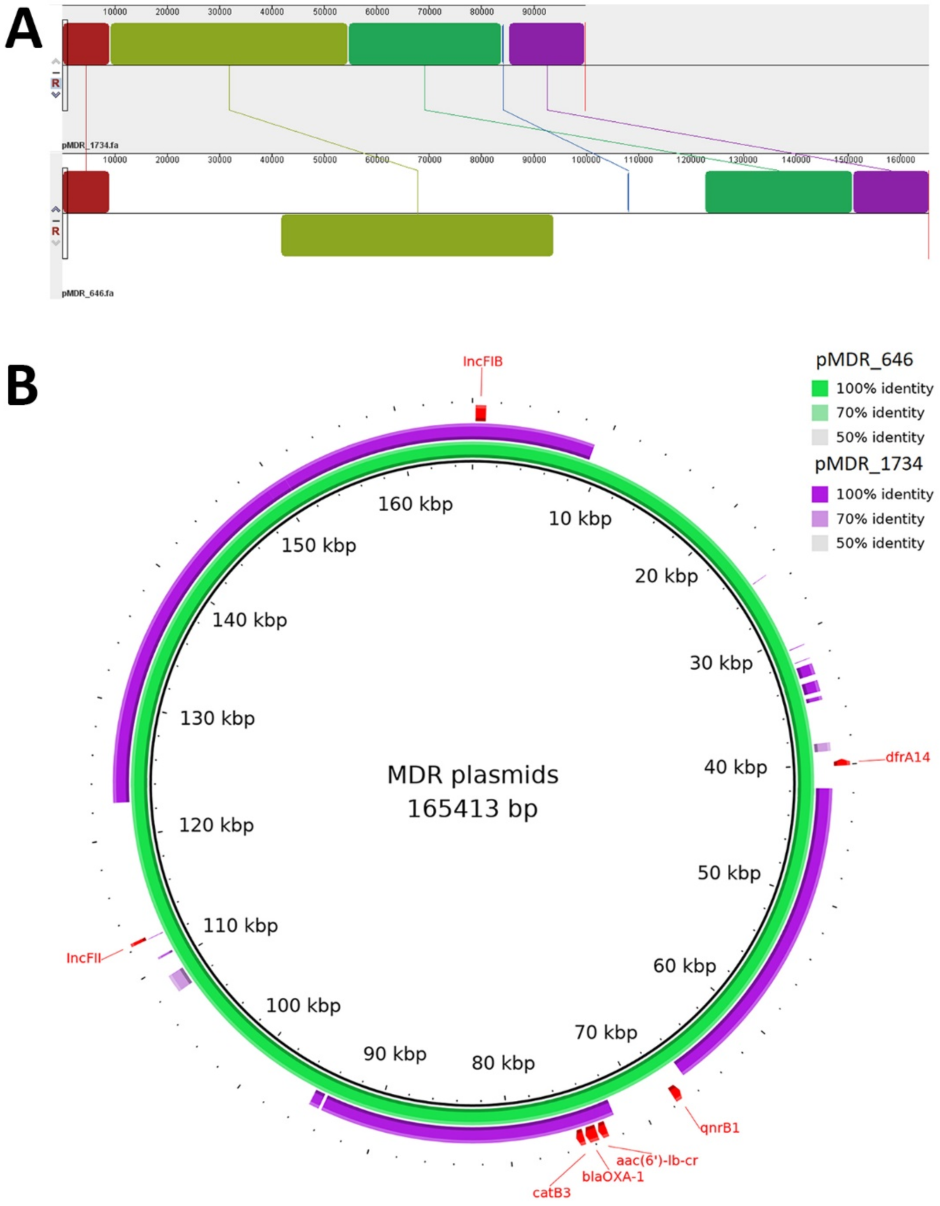

Appendix Figure. A) Alignment of pMDR_646 and pMDR_1734. The alignment was performed using Mauve. B) Feature-comparison of pMDR_646 and pMDR_1734. The map was generated using BRIG-0.95. MDR, multidrug resistance.
